# Prevalence and Characteristics of Patients Requiring Surgical Reinterventions for Ankle Fractures

**DOI:** 10.3390/jcm12185843

**Published:** 2023-09-08

**Authors:** Abraham Reyes-Valdés, Mirna Martínez-Ledezma, David Fernández-Quezada, José Guzmán-Esquivel, Martha Irazema Cárdenas-Rojas

**Affiliations:** 1 Hospital General de Zona No. 1, Instituto Mexicano del Seguro Social, Av. Lapislázuli No. 250 Colonia La Haya, Villa de Álvarez, Colima 28984, Mexico; dr.reyes.abr@gmail.com (A.R.-V.); mirnamale21@gmail.com (M.M.-L.); 2 Centro Universitario de Ciencias de la Salud (CUCS), Laboratorio de Microscopía de Alta Resolución, Departamento de Neurociencias, Universidad de Guadalajara, Guadalajara 44340, Mexico; david.fernandez@academicos.udg.mx; 3 Unidad de Investigación en Epidemiología Clínica, Instituto Mexicano del Seguro Social, Av. Lapislázuli No. 250 Colonia La Haya, Villa de Álvarez, Colima 28984, Mexico; pepeguzman_esquivel@outlook.com

**Keywords:** ankle, bone fracture, surgical traumatology, Danis–Weber

## Abstract

(1) Background: Ankle fractures are common injuries that typically require surgical treatment. Complications may arise, leading to reinterventions with poor recovery and reduced quality of life for patients. The aim of this study was to determine the number of patients who underwent surgical reintervention for ankle fractures, characteristics, and associated factors. (2) Methods: A cross-sectional study was conducted to analyze the number of patients requiring surgical intervention for ankle fractures at General Hospital Zone No1 IMSS in Colima over a period of two years. The age, gender, comorbidities, laterality, cause of surgical reintervention, Weber classification, and elapsed time to reintervention were analyzed. (3) Results: A total of 33 patients were included in this study, of whom 63.3% were male, ranging in age from 18 to 51 years old. The predominant Danis–Weber classification for both sexes was suprasyndesmotic fracture (Type C). No established relationship was found between comorbidities and surgical reintervention; however, a significant relationship was observed between home accidents and the need for reintervention. (4) Conclusions: Reintervention in patients previously operated on for ankle fractures is more frequent in male patients and those who sustained the injury at home.

## 1. Introduction

Ankle fractures are a common type of injury, with an incidence of 187 per 100,000 adults per year. Women are more prone to suffer ankle fractures in advanced ages, while in men, they occur in youth [1]. However, they occur more frequently in patients with osteoporosis, peripheral arterial disease, and diabetes mellitus; the latter present three times more postoperative complications [2]. Among ankle injury cases treated in the emergency department, 15% present fractures. In Mexico, a total of 3755 ankle fracture surgeries were performed in one year [3].

The ankle is stabilized with three groups of ligaments: the lateral collateral ligament complex, the syndesmotic ligament complex, and the medial collateral ligament complex (deltoid) [4]. During trauma, the ankle can be in two different positions: pronation (eversion) and supination (inversion). Additionally, three deforming forces can occur, abduction, adduction, and external rotation, which determine the four mechanisms of injury: pronation-abduction, pronation-external rotation, supination-adduction, and supination-external rotation [5,6]. Ankle fractures are often the results of sustained torsion, typically due to low-energy injury. The position of the ankle at the time of injury and the posterior direction of force typically determines the fracture pattern [4,7,8].

The Lauge–Hansen classification describes the position of the foot at the time of injury and the deforming force on the ankle, providing additional information about stability and the necessary treatment [9,10]. The Danis–Weber classification system classifies ankle fractures based on the location of the distal fibular fracture in relation to the syndesmosis. This classification divides fractures into three groups: type A (below the level of the syndesmosis, type B (at the level of the syndesmosis, and type C (above the syndesmosis) [11]. Weber type A can be treated conservatively, while Weber B and C are usually treated with surgery [4]. Also, an additional classification based on fracture stability is employed, wherein a unimalleolar fracture is regarded as stable and amenable to conservative management, while bimalleolar and trimalleolar fractures are deemed unstable and require surgical intervention [12].

The purpose of surgery for an ankle fracture is to restore the anatomical congruence of the ankle joint. When achieving this anatomical relationship is not possible, altered loading occurs in the tibiotalar joint, leading to poor outcomes. The type of surgery to be performed for fracture reduction and fixation depends on the type of fracture and the characteristics of the patients [4]. In cases where the fracture is not reduced, there is a risk of vascular complications, ischemia, joint damage, and prolonged inflammation of the ankle’s soft tissues, which could result in chronic pain. These unstable fractures are treated via open reduction and internal fixation in an operating room [7,8]. Despite the abundance of evidence to guide surgical management and achieve optimal outcomes in ankle fractures, results are often suboptimal [13]. The most frequent postoperative complications in the short term include wound hematoma and wound-edge necrosis, compartment syndrome, compromised wound healing, infection (reported in 2% of the patients), dislocation, malpositioned screw, inadequate reduction, and Complex Regional Pain Syndrome. In the mid to long term, patients may experience non-union, malposition, impingement syndrome, a restricted range of motion, and, in 10% of the cases, ankle arthrosis, with many necessitating reoperation [4].

Reported indications for performing surgical reintervention include problems with syndesmosis reduction and fibular shortening [14].

Although there are reports of complications following ankle fracture surgery, the frequency of this requirement is poorly documented. Based on previous reports, we anticipate that less than 29% of the patients will necessitate reintervention [15].

The aim of this study was to determine the number of patients requiring reintervention after open reduction with internal fixation of the ankle, as well as their characteristics and associated factors.

## 2. Materials and Methods

A cross-sectional retrospective study was conducted at General Hospital Zone No. 1, located in the western region of México, from 1 March 2018 to 31 December 2020. A comprehensive search was conducted in the INTQX database to identify all patients who underwent ankle fracture surgery. The patients undergoing ankle surgery presented a fracture displacement greater than 2 mm. The performed surgery was an open reduction with internal fixation using a plate for fractures with long lines or with multifragmentation, and screws were used for fractures with simple lines. After surgery, wound care was performed on patients, and they were administered antibiotics, analgesics, and anti-inflammatory drugs. The following day joint mobilization without weight-bearing was initiated. The patients had their sutures removed 15 days post-surgery, followed by radiographic assessment. Subsequently, they were examined every month for three months, then at sixth month, and went through a final review one year after the surgery. The criteria for discharging a patient following ankle fracture surgery are that they exhibit ranges of motion greater than 80% and do not experience incapacitating pain.

**Figure 1:**
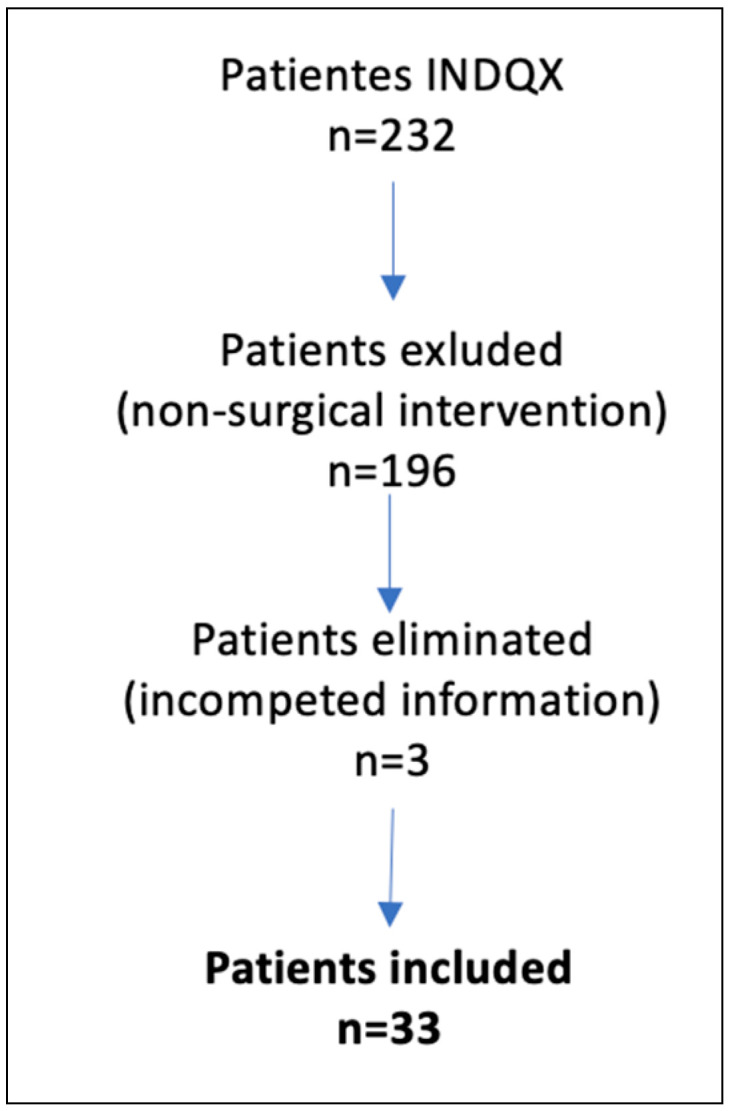
Flowchart for patients’ inclusion.

We enrolled all eligible patients meeting the predefined inclusion criteria, which encompassed individuals who underwent a surgical reintervention, defined by the necessity for additional surgical procedures for the ankle fracture. This study encompassed both sexes, individuals aged 18 years or older, and those fulfilling the Danis–Weber classification for ankle fracture diagnosis. Patients who underwent surgical reintervention to remove osteosynthesis material in cases of consolidated ankle fracture were excluded, as well as those in whom an osteosynthesis screw was removed under appropriate conditions. Additionally, patients whose initial surgery was performed at another medical facility were excluded. Patients with incomplete medical records were also eliminated from the analysis. Figure 1 shows the flowchart for patient inclusion.

All patients who met the inclusion criteria were selected by sampling for convenience.

Information on age, gender, comorbidities (diabetes mellitus, high blood pressure, hyperuricemia, and chronic renal disease), laterality, cause of surgical intervention, Weber classification, and time elapsed until reintervention was obtained using the Electronic Medical Record and the Official Bed Information System, which are the electronic platforms used to store all patient’s information for outpatient consultation and hospitalizations, respectively.

Descriptive statistical analysis was performed using measures of central tendency, standard deviation, frequencies, and percentages for categorical variables, while the chi-square test was used for inferential analysis, considering statistical significance at a *p*-value < 0.05. The software used for the analysis was SPSS V.25

This study was approved by de Local Research Committee-601, with registration number R-2021-601-041.

## 3. Results

### 3.1. Characteristics of the Population

A total of 232 patients with ankle fractures underwent surgical intervention, among whom 33 individuals (14%) necessitated surgical reintervention and met the inclusion criteria. Table 1 presents the characteristics of the study population.

### 3.2. Diagnosis and Treatment of Ankle Fracture

According to the Weber classification, the majority of patients requiring surgical reintervention had a higher frequency of Weber C classification at 81.8% (n = 27), followed by Weber B classification at 18.2% (n = 6). 1. Additionally, among the patients who required surgical reintervention, 16 (48.3%) presented with unimalleolar fractures, 7 (21.1%) had bimalleolar fractures, 9 (27.3%) had trimalleolar fractures, and 1 (3%) exhibited pseudoarthrosis.

A total of 27 patients (82%) exhibited syndesmosis instability and were subject to positioning screw and plate placement. One patient (3%) received a plate and suture on the medial malleolus, two patients (6%) underwent sole plate fixation, and three patients (9%) received medial malleolar screw placement.

When analyzing the time interval between the first open reduction with internal fixation and the surgical reintervention for ankle fracture, a wide variation was observed, ranging from 1 to 1095 days. The mode was 3 days, corresponding to 12 cases (36.4%). Table 2 presents all the causes that required surgical reintervention for ankle fractures.

### 3.3. Factors Associated with Surgical Reintervention

It was determined that age is not associated with the type of fracture and the need for surgical reintervention, as shown in Table 3.

The association between gender and ankle fracture type in patients undergoing surgical reintervention is shown in Table 4. Despite the fact that gender is not associated with surgical reintervention, a higher frequency is observed among male individuals.

No association was observed between fracture type and comorbidities in patients undergoing surgical reintervention, as shown in Table 5.

A statistically significant association was found between the type of accident and ankle fracture, with a *p*-value of 0.014. Home accidents showed an association with the need for surgical interventions in patients who had unimalleolar fractures. The details of this association are presented in Table 6.

## 4. Discussion

A surgical reintervention was required in 14% of the patients, which is slightly lower than the previously reported [15]. This discrepancy could be attributed to differences in study design and the inclusion criteria utilized. It was observed that the most frequent group of patients undergoing reintervention comprised individuals aged between 18 and 51 years [13], with males being the most common [16]. This coincides with previous findings that also reported a higher frequency of ankle fractures in this age group, possibly due to the occupational and sports activities they engage in. The fact that this group is more prone to ankle fractures also predisposes them to require more surgical reinterventions.

Regarding weight, an average of 70 kg was found, considered within the normal range, and no association was found between weight and the need for surgical reintervention in our study, as previously reported [17].

However, other reports indicate that diabetes mellitus and the high body mass index are risk factors for open reduction with internal fixation of the syndesmosis in ankle malleolar fractures, as well as worse outcomes and poor functional results [2,18,19]. These differences could be due to variations in study design and sample size, which are larger than in previous studies. Similar to this study, it has been previously reported that 64% of ankle fractures occur due to accidents during domestic or everyday activities, so this cause should not be underestimated [20]. Regarding laterality, a higher frequency of fracture was observed on the right side, in line with previous findings that investigated postoperative complications and reoperation rates in ankle fractures. In their results, they found that 51.2% of the reinterventions were classified as Danis–Weber C [21]. When analyzing comorbidities, it was found that type 2 diabetes mellitus is the most common disease among patients undergoing reintervention for ankle fracture, although this association was not statistically significant. However, diabetes mellitus has effects on fracture risk and is associated with higher morbidity compared to the general population. A higher risk of fractures has been reported in patients with diabetes mellitus, including an increased risk of lower extremity fractures and other fractures [22]. The presence of diabetes mellitus in patients with ankle fractures can complicate surgery and postoperative recovery, even with the care of an experienced surgeon. Although evidence on surgical management and decision-making in ankle fractures in diabetic patients is limited, it has been shown that immediate surgical intervention is appropriate in closed ankle fractures in patients with decompensated diabetes mellitus type 2 prior to operation [23,24].

Regarding the time interval between open reduction and internal fixation and surgical reintervention in ankle fracture patients, it was found to be 3 days. This result differs from a previous study where most intervened patients returned to the hospital eight weeks or later after the initial surgery, which was one of the inclusion criteria [25,26].

Regarding the causes of surgical reintervention, inadequate syndesmosis closure was found to be the most frequent cause. The authors agree that syndesmosis injuries require surgical treatment, as their integrity is essential for maintaining normal movement. An unresolved injury to this structure can lead to various complications, including arthritis.

The limitations of this study include its retrospective design and relatively small sample size, which restricted the analysis to a specific population without considering the entire cohort of patients who underwent ankle surgery.

## 5. Conclusions

Reintervention in patients previously operated for ankle fractures is more frequent in male patients and those who sustained the injury at home.

## Figures and Tables

**Table 1:** Characteristics of the study population.

	n = 33 Patients	Measure
***Age (years)***		
18–35	11	33.3 *
36–51	11	33.33 *
52–68	9	27.3 *
69–85	2	6.1 *
***Gender***		
Male	21	63.6 *
Female	12	36.4 *
***Weight (years)***		
18–35	11	74.6 +
36–51	11	71.13 +
52–68	9	74.0 +
69–85	2	62.0 +
***Side of injury***		
Left	11	33.3 *
Right	22	66.7 *
***Injury cause***		
Activities at home (fall or blow)	15	45.5 *
Sport	4	12.1 *
Traffic accidents	10	30.3 *
Activities at work	4	12.1 *
***Comorbidity***		
Hyperuricemia	1	3 *
Type 2 diabetes mellitus	5	15.2 *
High blood pressure	2	6.1 *
Chronic kidney disease	1	3 *
None	24	73.7 *

* Percentage. + Kilograms.

**Table 2:** Causes of surgical reintervention in patients with ankle fractures.

	Frequency (n)	Percentage (%)
Osteoarthritis and residual ankle deformity	1	3
Osteoarthritis and ankle pain + talar necrosis	1	3
Displaced fragments/ankle instability	4	12.1
Inadequate closure of the syndesmosis	7	21.2
Inadequate closure of the medial clear space	1	3
Dislocated medial clear space	1	3
Ankle pain/wound exudate	1	3
Pain/exposure to osteosynthesis material	1	3
Lack of mobility due to inadequate rehabilitation	1	3
Osteosynthesis material fatigue	1	3
Fistula + exposure to osteosynthesis material	2	6.1
Surgical wound infection	1	3
Intolerance to osteosynthesis material	1	3
Intolerance to osteosynthesis material + fistula	1	3
Insufficient material, displacement of fracture fragments	2	6.1
Osteomyelitis + rejection of osteosynthesis material	1	3
Left ankle osteomyelitis	1	3
Ankle nonunion fracture	1	3
Inadequate reduction in lateral malleolus	1	3
Displaced fracture line	3	9.1

**Table 3:** Analysis of the association between age and fracture type in patients undergoing surgical reintervention.

Age (Years)	Unimalleolar Fracture (n)	Bimalleolar Fracture (n)	Trimalleolar Fracture (n)	Non-Union (n)	*p* Value *
18–35	4	4	2	1	0.328
36–51	7	0	4	0	
52–68	3	3	3	0	
69–85	2	0	0	0	
Total	16	7	9	1	

* Chi-square.

**Table 4:** Association between gender and fracture type in patients undergoing surgical reintervention for ankle fracture.

Gender	Unimalleolar Fracture (n)	Bimalleolar Fracture (n)	Trimalleolar Fracture (n)	Non-Union (n)	*p* Value *
Female	5	4	3	0	0.553
Male	11	3	6	1	
Total	16	7	9	1	

*Chi-square.

**Table 5:** Association between comorbidities and fracture type.

Comorbidities	Unimalleolar Fracture (n)	Bimalleolar Fracture (n)	Trimalleolar Fracture (n)	Non-Union (n)	*p* Value *
Hyperuricemia	1	0	0	0	0.428
Type 2 diabetes mellitus	2	1	2	0	
High blood pressure	0	2	0	0	
Chronic kidney disease	0	0	1	0	
None	13	4	6	1	

* Chi-square.

**Table 6:** Association between the type of accident and fracture type in patients undergoing surgical reintervention.

Accident	Unimalleolar Fracture (n)	Bimalleolar Fracture (n)	Trimalleolar Fracture (n)	Non-Union (n)	*p* Value *
Sport	4	0	0	0	0.014
Home	9	3	3	0	
Work	0	0	3	1	
Traffic	3	4	3	0	

* Chi-square.

## Data Availability

No new data were created or analyzed in this study. Data sharing is not applicable to this article.
